# Expression analysis of cellulose synthase-like genes in durum wheat

**DOI:** 10.1038/s41598-018-34013-6

**Published:** 2018-10-23

**Authors:** Ilaria Marcotuli, Pasqualina Colasuonno, Antonio Blanco, Agata Gadaleta

**Affiliations:** 1 Department of Agricultural and Environmental Science, University of Bari ‘Aldo Moro’, Via G. Amendola 165/A, 70126 Bari, Italy; 2 Department of Soil, Plant and Food Sciences, University of Bari ‘Aldo Moro’, Via G. Amendola 165/A, Bari, Italy

**Keywords:** Gene expression, DNA sequencing

## Abstract

Cellulose synthase-like *CslF* and *CslH* genes have been implicated in the biosynthesis of β-glucans, a major cell wall constituents in grasses and cereals. The low β-glucan content of durum wheat and lack of information of the biosynthesis pathway make the expression analysis in different developmental stages of grain endosperm an interesting tool for the crop genetic improvement. Specific genome sequences of wheat *CslF6* and *CslH* were isolated and the genomic sequence and structure were analysed in the cv. Svevo. In starchy endosperm at five developmental stages (6, 12, 21, 28 and 40 days after pollination) *CslF6* and *CslH* transcripts were differentially expressed. A peak of *CslF6* transcription occurred at 21 dap, while *CslH* was abundant at 28 dap. Significant variations were detected for both the genes in the genotypes. Significant and positive correlation were detected between β-glucan content and *CslF6* gene expression at 21 dap and 40 dap, while no significant correlation was observed for *CslH* gene. On the overall, our correlation analysis reflected data from previous studies on other species highlighting how the abundance of transcripts encoding for CslF6 and CslH enzymes were not necessarily a good indicator of enzyme activity and/or β-glucan deposition in cell wall.

## Introduction

Wheat is one of the most important cereal in the world, providing nutrients for humans and animals. Non-starch polysaccharides (NSP) are important components of wheat kernels and many studies have shown that they have health benefits, including immunomodulatory activity, cholesterol lower activity, faecal bulking effect, enhanced absorption of certain mineral, prebiotic effects, and reducing type II diabetes^1^. Recently, the attention on food quality over quantity is growing in many areas of the world and dietary fibre are gaining much interest. The principal components of the NSP are β-glucans and arabinoxylans. Even though β-glucans are a minor component of wheat cell walls, they are one of the most important portion of the soluble fibre fraction with beneficial effects for human health.

As for many plant polysaccharides, how and where β-glucans are synthesized, which genes are involved and specific functions, interactions and activities of each enzyme are not totally available^2^. A superfamily of genes are involved in the synthesis of these polysaccharides, which include *cellulose synthase* (*Ces*)^2–5^ and *cellulose-synthase-like* (*Csl)* families^6^. It has been demonstrated that the superfamily *Csl* is responsible for the synthesis of several plant cell wall polysaccharides and includes subfamilies from A to H, each of which consists of multiple genes^7^. For example, in rice (*Oryza sativa* L.) there are 37 *Csl* genes in total, while in *Arabidopsis* there are 30^8,9^. Not all *Csl* subfamilies are represented in higher plant groups. Thus, the *CslB* and *CslG* subfamilies are found only in dicotyledons and gymnosperms, whereas the *CslF* and *CslH* groups are found only in monocotyledons^10^, which regulate directly or indirectly the abundance and the fine structure of β-glucans in both grain and the other part of the plant^11^.

Burton *et al*.^11^ reported that the over-expression of a *CslF* gene, under the control of an endosperm-specific promoter, resulted in the increase of β-glucan content and a dramatic decrease of starch in the transgenic grain. *Brachypodium distachyon*, which has a β-glucan content over 40% of the grain weight and the starch about 6%, provides additional support for a regulatory link between starch and β-glucan synthesis^12^. Furthermore, when Arabidopsis was transformed with the *OsCslF6* gene, mixed-linkage glucan was detected in the cell wall indicated that *CslF6* was capable of synthesizing beta glucan^6,13^. Four of the corresponding *CslF* genes of barley were mapped to a locus on chromosome 2H (*HvCslF3, HvCslF4, HvCslF8, HvCslF10*) and two other genes on chromosomes 1H (*HvCslF9*) and 7H (*HvCslF6)* corresponding to quantitative trait loci (QTL) for grain *β*-glucan content^14^.

In rice, *OsCslF6* knockout mutants synthesizes low amount of β-glucan^15^. Nemeth *et al*.^16^ identified the *CslF6* gene of wheat and demonstrated that transgenic manipulation by iRNA modify the amounts and properties of *β*-glucan in wheat. Other studies showed how the addition of barley chromosome 7H (on which *HvCslF6* is located) in wheat genome increases the *β*-glucan production^17,18^.

Members of the *CslH* gene family have also been shown, by transformation into *Arabidopsis*, to be capable of beta glucan synthesis^13^. The barley *HvCslH* expressed into transgenic *Arabidopsis* lines determined a higher amount of CslH protein accumulated in the walls^13^. In addition, *HvCCslH* gene resulted associated to a QTL for β-glucan amount on barley chromosome 2H and expressed in various stages of grain development^19^.

Due to the complex biosynthetic mechanism, in order to provide an efficient and correct synthesis of β-glucans, one or more additional proteins interacting with *CslF* and *CslH* enzymes are probably required^20^. The availability of sequences for *CslF* and *CslH* gene family members allowed the study of the transcript abundance in developing barley grain. At about eight days after pollination (dap), when cellularization of developing endosperm is essentially complete^21^, there is a transient increase in *CslF9* transcripts; these subsequently decrease to very low levels by 16 dap^14^. The levels of *CslF6* transcripts are high throughout endosperm development and increase during the latter stages of grain maturation^14^. Transcripts of the *CslH* genes remain low throughout endosperm development in barley, but this is not to dismiss them as unimportant in β-glucan biosynthesis^13,14^. The low amount of β-glucan content in durum wheat kernel and the lack of information of the biosynthetic pathway make the biosynthesis and accumulation studies fundamental for the molecular breeding. In fact, the *CslF* and *CslH* gene families have not yet been compiled in wheat. For this reason, we present the study of the *CslF6* and *CslH* transcription pattern in ten durum wheat cultivars characterized by different β-glucan content. The aim of our work was to characterize the gene sequences of the two genes (*CslF6* and *CslH*) and correlate the gene expression with the final β-glucan content in wheat grains.

## Results and Discussion

The main causes of morbidity and mortality in affluent, developed economies are colorectal cancer, cardiovascular disease, and diabetes^22^. Dietary fibre reduces the risk of contracting these serious human diseases and reduces the adverse social and personal condition impact.

The strongest evidence for the contribution of dietary fibre to disease prevention comes from the European Prospective Investigation into Cancer and Nutrition (EPIC), which shows a very strong and dose-dependent reduction in the risk of colorectal cancer^1^, of obesity^1,23,24^, diabetes^25^, diverticular disease^26^, and cardiovascular disease^25^ with greater dietary fibre consumption. Some of the effects of dietary fibre are due to the insoluble non-starch polysaccharides such as β-glucan.

Wheat is not recognized as a significant source of β-glucan because of it has <1% on a dry weight basis content^27^. Despite this, products such as wheat bran are extremely effective at relieving constipation through a stool bulking effect.

The biosynthetic pathway of β-glucan has been deeply studied in barley^6,11,13,14^, but limited data are reported for durum wheat^16^. For this reason, we report the A and B genome sequences of the two main genes, *CslF6* and *CslH*, involved in the β-glucan biosynthesis in durum wheat, and the expression study carried out in the endosperm at different developmental stages to clarify the transcription pattern of those two genes and the linkage with fibre accumulation.

### Isolation of genomic sequences of *Csl* genes in durum wheat

The sequences corresponding to *CslF6* and *CslH* genes from *Oryza sativa* (Os08g06380 and Os04g35030, respectively) and *Hordeum vulgare* (MLOC_57200 and MLOC_53007, respectively) were used as starting point to isolate the durum wheat sequences. As reported in Supplementary File S1, using the cv. Svevo, the A and B homoeologous sequences of the *Csl* durum genes were isolated with the corresponding cDNAs. Due to the availability of SNP data in bread and durum wheat, the current analysis allowed to detect some SNP markers inside both genes. In particular, two (IWB6109 and IWB23981) and four (IWB4561, IWB4622, IWB14446, IWB66376) SNP markers, corresponding to *CslF6* and *CslH* gene sequences, respectively, were identified. The SNPs within *CslF6* gene mapped on chromosomes of group 7, whereas those of the other gene localized on chromosome group 2 (Table 1).

**Table 1:** List of β-glucan genes in wheat with corresponding SNP markers, allele change and scaffold localization.

Gene	Enzyme	SNP name	SNP id	Allele	Scaffold
*CslF6*	Cellulose synthase-like protein F6	BS00009773_51	IWB6109	T/G	TGACv1_scaffold_592076_7BS: 95,657-95,721
		Excalibur_c23481_1065	IWB23981	T/C	TGACv1_scaffold_555973_7AL: 251,795-251,905 TGACv1_scaffold_577473_7BL: 37,700-37,810 TGACv1_scaffold_607937_7DL: 4,686-4,796
*CslH*	Cellulose synthase-like protein H	BobWhite_c90_1565	IWB4561	A/G	TGACv1_scaffold_158387_2DL: 95,998-96,108 TGACv1_scaffold_094351_2AL: 5,576-5,686
		BobWhite_c9557_347	IWB4622	A/G	TGACv1_scaffold_129372_2BL: 297,426-297,698
		CAP7_rep_c6934_84	IWB14446	T/C	TGACv1_scaffold_129372_2BL: 298,012-298,119
		Tdurum_contig102249_324	IWB66376	A/G	TGACv1_scaffold_158387_2DL: 94,798-94,905 TGACv1_scaffold_094351_2AL: 6,791-6,898

### Characterization of wheat *Csl* gene sequences

**Figure 1:**
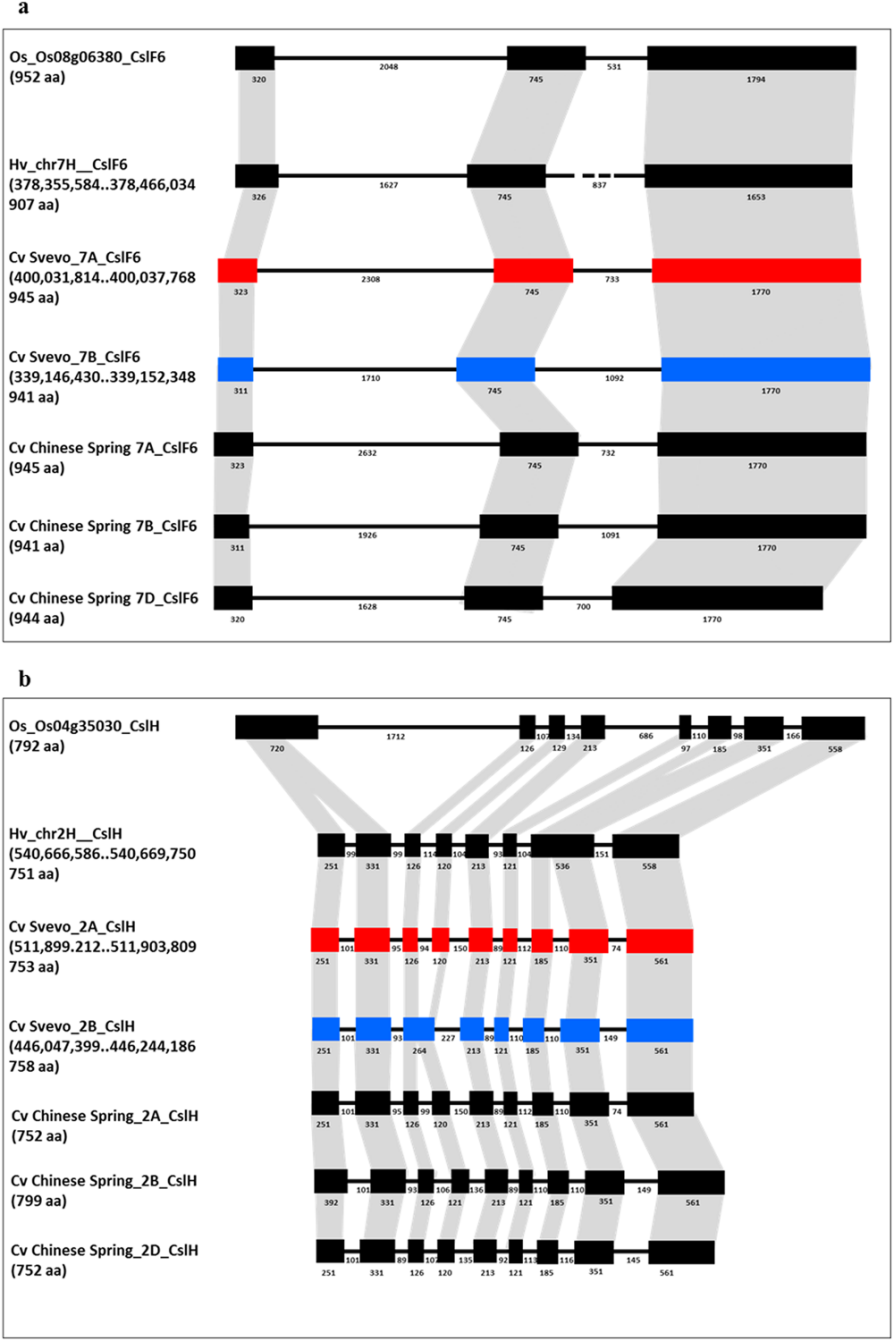
Comparison of *CslF6* (**a**) and *CslH* (**b**) gene structures in rice, barley, durum wheat (A and B genome) and bread wheat (A, B and D genome) is shown based on coloured boxes highlighting conserved exons. Intron and exon sizes are shown as well as the whole gene (in brackets the total length). Rice, barley and both wheat *CslF6* share the same structure with three exons of conserved sizes and two introns. Differences were detected in *CslH* structure among the three species. Black dashed line indicates the absence of intron sequence.

As our main interest was the study of the possible relationships between *Csl* genes and β-glucan accumulation in the wheat grain, we analysed the genomic sequence and structure of *CslF6* and *CslH* genes in the cv. Svevo (Fig. 1a,b). Relying on our bioinformatics analysis regarding the *CslF6-7A* gene, the genomic sequence was 5,879 bp, including an mRNA of 2,838 bp and a protein of 945 amino acids. For *CslF6-7B*, the genomic sequence length was 5,628 and allowed to obtain a cDNA of 2,826 bp codifying a protein of 941 amino acids.

Fgenesh^++^ gene prediction was used to define the intron/exons structure predicting a similarity between both the wheat *CslF6* genes composed by 3 exons and 2 introns sharing an identity of 97% among the homoeologues. Differences were underlined for the first exon between the A and B genome, while exons two and three displayed 100% of identity (Fig. 1a). BLAST analysis using Phytozome v.7 software (http://www.phytozome.net) with *O. sativa* and *H. vulgare* genomes allowed the comparison of wheat *CslF6* genes with the orthologous genes located on chromosome 8 of rice (locus name: Os08g06380) and chromosome 7 of barley (locus name: MLOC_57200). The rice *CslF6* had a sequence of 5,438 bp with a CDS of 2,859 bp, whereas the barley gene had a sequence of 5,188 bp with a CDS of 2,724 nucleotides. Considering the wheat *CslF6-7A* with the rice and barley genomic sequences the identities detected were 88% and 96%, respectively, and 96% between wheat cDNA and the other two CDS considered. A similar intron/exons structure was observed between *CslF6-7A* from wheat and *CslF6* from rice and barley composed by 3 exons and 2 introns (Fig. 1a). An identity of 88% were detected between wheat and rice *CslF6* genomic sequence and 87% between the two cDNA sequences. Whereas identity of 96% was found aligning both the *CslF6-7B* genomic sequence and mRNA with the barley *CslF6* ones.

Comparison between both the wheat CslF6 protein sequences (A and B genome) and rice and barley enzymes showed identity of 85% and 98%, respectively.

**Figure 2:**
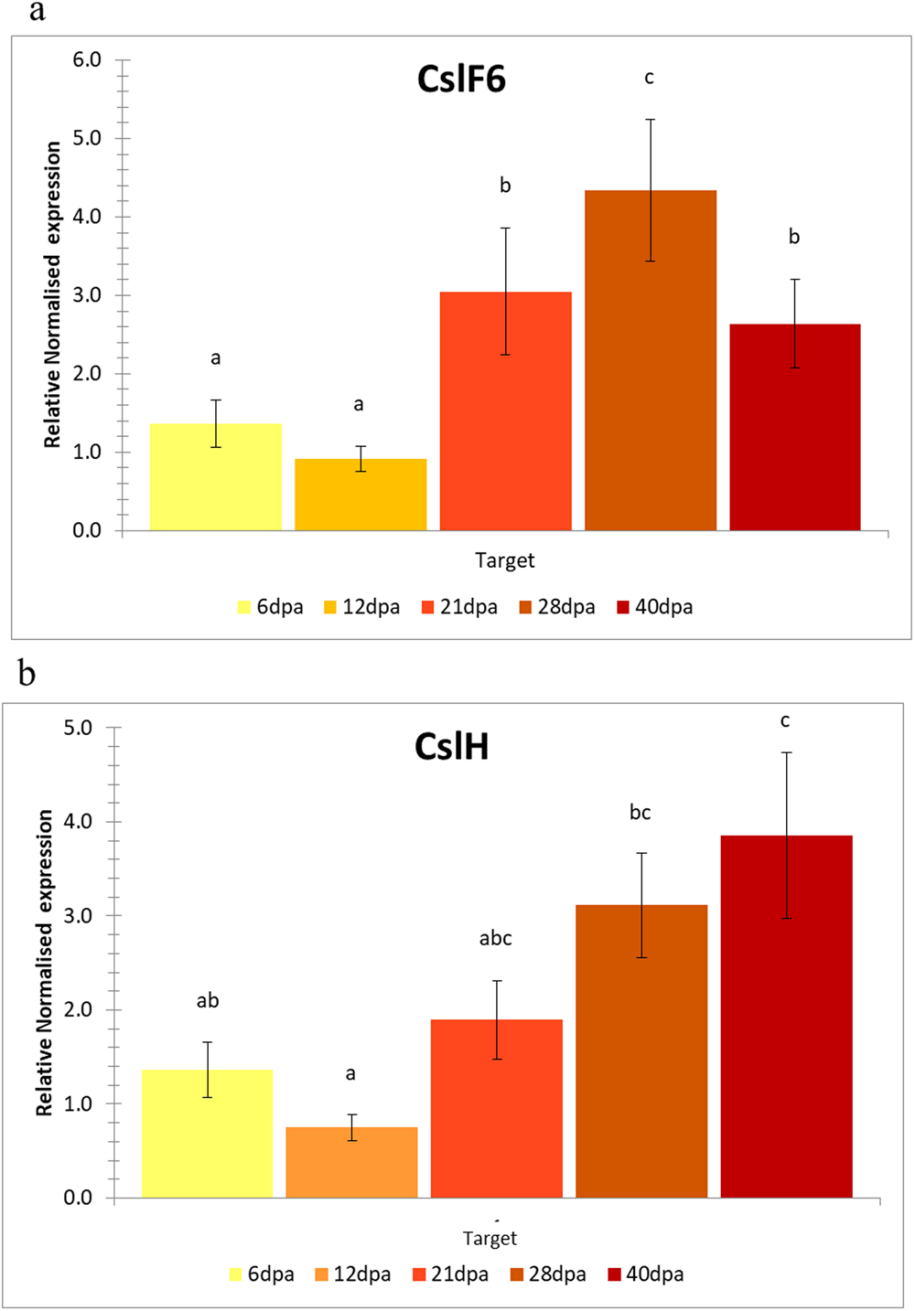
Gene expression study of *CslF6* (**a**) and *CslH* (**b**) in durum wheat endosperm at different developmental stages (6, 12, 21, 28 and 40 days after pollination). Different letters on the bars indicate datasets significantly different according to ANOVA followed by Duncan’s test (P < 0.05).

In addition, *CslF6* gene sequences from bread and durum wheat were compared (Fig. 1a) showing for both the A and B genome an identity of 99%. In details, Chinese Spring *CslF6-7A* had a genomic sequence of 5,879 bp and the same structure and exons length of durum wheat, while differences were detected into the introns length. Interesting results were observed for the bread B genome, which showed a gene of 5,843 bp with same exon number and length, different introns amino acid number and a transposome of 212 bp into the first intron, which could be one of the reason of a different and higher final β-glucan content in bread wheat. In addition, the transposome gene located in the first intron of the B gene is responsible of the synthesis of four different transcripts of 941 aa, 827 aa, 806 aa and 643 aa, respectively. Interesting results were detected for the *CslH* genomic sequence and structure. In details, the *CslH-2A* gene sequence was 3,089 bp, counting a cDNA of 2,259 bp and a protein of 726 amino acids, while the *CslH-2B* had a gene sequence length of 3,156 bp, transcribing a mRNA of 2,277 bp and a final protein size of 595 amino acids. Different intron/exons structure was detected between the two *CslH* homeologues: 9 exons and 8 introns were reported for the A genome gene and 8 exons and 7 introns for the B genome with 95% of identity. The difference among the intron/exon numbers is due to the merging of the exons 3 and 4 of the A genome into exon 3 of the B genome (Fig. 2b).

Again the *CslH* gene sequences from Chinese Spring and Svevo were compared highlighting same gene length of 3,089 bp and intron/exon structures for A genome. Three different transcripts were found for *CslH-2A* counting of 752 aa, 748 aa and 737 aa.

Differences were detected examining sequences from bread and durum B genomes. The Chinese Spring *CslH*, in fact, showed a gene of 3,295 bp, with a cDNA of 2,400 bp and a transcript of 799 with a gene structure similar to the A genome with 9 exons and 8 introns.

Valuation of wheat *CslH* genes with the orthologous genes located on rice chromosome 4 (locus name: Os04g35030.1) and barley chromosome 2 (locus name: MLOC_53007) was carried out. The rice and barley *CslH* had a sequence of 5,392 bp and 3020 bp, with a CDS of 1,218 bp and 2,256 nucleotides, respectively. Analysis of identity between wheat *CslH-2A* and *CslH-2B* and rice genes showed a same score for genomic and CDS sequences (84% and 79% respectively), while 66% and 63% for the proteins. Wheat *CslH-2A* and *CslH-2B* and barley genomic sequences showed identities of 90% and 87%, respectively, with 93% for both the considered CDS and 89% and 84% for the proteins.

A comparative analysis of the durum *CslH-2A* gene and the correspondent bread wheat one was not carried out due to the lack of bread gene portions, while identity of 100% was detected between the two wheat *CslH-2B* genes.

Different intron/exons structure was observed between wheat *CslH-2A* and rice *CslH* composed by 9 exons and 8 introns and 8 exons and 7 introns, respectively, due to the splitting of the rice first exon into two wheat exons (Fig. 1b). Barley *CslH* structure showed a merge of exon 7 and 8 of durum *CslH-2A* into barley *CslH* exon 7. The *CslH-2B* showed the same intron/exon numbers of rice and barley *CslH* with 8 exons and 7 introns, but in this case rice exon 1 corresponded to wheat exons 1 and 2 and rice exons 2 and 3 merged into wheat exon 3; however barley exon 3 and 4 combined in wheat exon 3, and barley exon 7 split in wheat exons 6 and 7 (Fig. 1b).

### β-glucan content and expression profile of *CslF6* and *CslH* genes

The *CslF6* and *CslH* gene expression levels were determined in eight durum wheat genotypes (Avonlea, Canyon, Cappelli, Ciccio, Duilio, Latino, Simeto, Svevo) and in two ssp. *dicoccoides* accesions (MG4328/61, MG4413).

Total β-glucan amount for each line in three different years with LDS and coefficient of variation was reported in Table 2. The ANOVA analysis revealed highly significant variation (P ≤ 0.001) among genotypes (Table 3). Avonlea showed the highest β-glucan content (0.70%) followed by Canyon (0.62%), while MG4413 had the lowest amount (0.28%). Our results were in line with what previously reported^28–30^, in fact, wheat is not recognized as a significant source of β-glucan because of it has low content in the grain, usually <1% on a dry weight basis^28^. Although values up to 2.3% have been reported in bread wheat^27^. The significant concentration of β-glucan in wheat grain is in the sub-aleurone layer, while low amount was found in the rest of the endosperm^31,32^. Because the wheat endosperm is ground into flour and provides nutrition in the form of starch and proteins, kernels with higher β-glucan content would make durum wheat a good and complete source of nutrients for human diet.

**Table 2:** β-glucan content of ten durum wheat cultivar grown at Valenzano (Metropolitan City of Bari, Italy) for three years (2012, 2013 and 2016). The reported value are the mean of three biological replicates. LSD and coefficient of variation were calculated in GenStat 14.

Line	β-glucan (% w/w)		
	Valenzano 2012	Valenzano 2013	Valenzano 2016
Avonlea	0.68	0.70	0.70
Canyon	0.65	0.60	0.62
Simeto	0.46	0.46	0.46
Ciccio	0.45	0.47	0.46
Latino	0.47	0.44	0.46
Duilio	0.44	0.39	0.42
Cappelli	0.39	0.40	0.40
Svevo	0.41	0.25	0.33
MG4413	—	0.28	0.28
MG4328	0.50	0.41	0.46
LSD	0.09	0.10	0.12
CV (%)	8.54	3.50	4.32

**Table 3:** Mean squares from the analysis of variance of β-glucan content in ten wheat genotypes grown at Valenzano (Bari, Italy) in three years.

Source of variation	Degrees of freedom	Mean square
Blocks	2	0.002
Year	2	0.017
Genotype	9	0.081***
G × Y	17	0.003
Error	28	0.003

***Significant differences P ≤ 0.001.

To better understand the wheat β-glucan biosynthesis and accumulation, we analysed the expression pattern of the two main genes, *ClsF6* and *CslH*, associated with β-glucan concentration in barley^6,11,13^. Starting from ten genotypes, our aim was to define the expression pattern of the two genes and highlight a possible correlation with final β-glucan kernel content. mRNA extracted from endosperm of the ten lines at different developing endosperm stages (6, 12, 21, 28 and 40 days after pollination) was used to determine the transcription levels of *CslF6* and *CslH*. Considering the mean values of the ten genotypes, we observed that the expression pattern of the two genes was different. Levels of *CslF6* mRNA were generally low in the first two developmental stages (6 and 12 dap) and relatively abundant in endosperm at 21 and 28 dap (Fig. 2a), while *CslH* transcription levels, less expressed compared to *CslF6* transcripts, were low at 6 and 12 dap, moderately high at 21 dap and high at 28 and 40 dap (Fig. 2b). Our data are supported by previously studies in barley, which reported variation on the transcription levels of *HvCslF6* throughout endosperm development^21^ with increases in the abundance of expression from 12 to 20 dap^14,29^. Different results were reported in barley for *HvCslH* transcripts. Maximum transcript levels, in fact, were reached at 4 dap and slightly amount of *HvCslH* were detected at 24 dap^13^. The different data of *HvCslH* transcripts between our report and previously studies in barley could be due to two different reasons: the endosperm developmental stages used for the experiments (we analysed the expression pattern until 40 dap, while in barley from 4 to 24 dap) and the role of this gene in β-glucan synthesis during secondary wall development in the two different species (wheat and barley)^33^.

**Figure 3:**
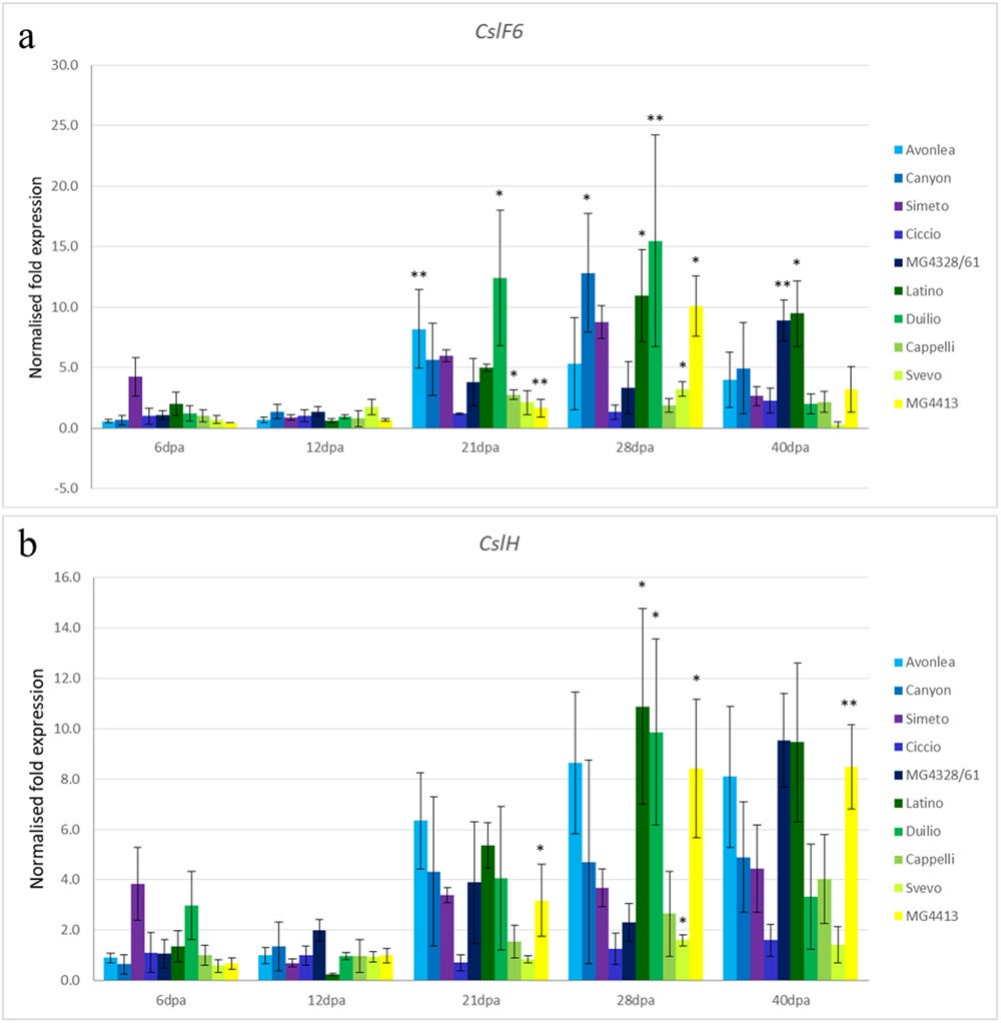
Normalized expression levels for the wheat *CslF6* (**a**) and *CslH* (**b**) genes in developing endosperm at various times (days) after the initiation of maturation (6, 12, 21, 28 and 40 days after pollination) analysed in ten durum wheat lines. Data represent average values obtained for three independent lines. Asterisks indicate genotypes significantly different within the developmental stages (**P ≤ 0.01; *P ≤ 0.05).

The abundance of transcript of *CslF6* in each genotypes was monitored independently during the development of wheat endosperm. As shown in Fig. 3 the genotypes showed different gene expression amount in the developmental stages analysed. The varieties, with higher transcript variation, resulted, for both the genes, Avonlea, Canyon, Latino, Duilio and MG4413. Transcripts of the *CslF6* gene appeared statistically significant (P ≤ 0.01; P ≤ 0.05) in Avonlea, Duilio, Cappelli and MG4413 at 21 dap; Canyon, Latino, Duilio, Svevo and MG4413 at 28 dap; MG4328/61 and Latino at 40 dap (Fig. 3a). Previous studies^13–19,34^ on differential expression of cellulose synthase-like genes *CslF6* in grain developmental stages confirmed our data on wheat kernel, detecting differences between barley lines during the endosperm developmental stages.

Again, differences between the ten varieties were detected for the *CslH* expression amount (Fig. 3b) as reported in literature on barley varieties^19^. In detail, MG4413 showed statistical significant difference (P ≤ 0.05) at 21 dap; Latino, Duilio, Svevo and MG4413 at 28 dap ((P ≤ 0.05); and MG4413 at 40 dap (P ≤ 0.01).

The second aim of our work was to correlate the gene expression with the final β-glucan content in grains. Correlation analysis between *CslF6* and *CslH* transcripts and β-glucan at each developmental stage were implemented in GenStat (Table 4). Significant and positive relationships were observed among β-glucan content and *CslF6* expression at 21 dap and 40 dap (P ≤ 0.01 and P ≤ 0.05, respectively). The data observed were in line with what reported by Burton *et al*.^14^ in two barley varieties, the elite malting variety and the hulless barley.

**Table 4:** Correlation analysis between gene expression and β-glucan content at different developmental stages.

Genes		Gene expression				
		6 dap	12 dap	21 dap	28 dap	40 dap
*CslF6*	BG	−0.062	−0.099	0.835**	0.385	0.670*
*CslH*	BG	−0.035	0.171	0.628	0.143	0.192

**Significant differences P ≤ 0.01.

*Significant differences P ≤ 0.05.

Our experiment did not allowed us to find any significant correlations between β-glucan content and the expression of the *CslH* gene during the endosperm maturation. Even other results reported in literature on barley grain^13–19^ highlighted how levels of *HvCslH* transcripts were relatively low throughout the starchy endosperm during development compared to other genes involved into β-glucan biosynthesis, confirming the complexity of this trait and the necessity of further investigation at the enzymatic level and localization of gene expression.

On the overall, our correlation analysis reflected data from previous studies on other species^6,11,13,14^ highlighting how the abundance of transcripts encoding for CslF6 and CslH enzymes were not necessarily be a good indicator of enzyme activity and/or β-glucan deposition in cell wall. In addition, the molecular mechanisms of β-glucan biosynthesis is complex and different enzymes are crucial for their deposition and accumulation^19^.

## Material and Methods

### Plant material

A set of ten genotypes, including eight cultivars of durum wheat, *Triticum turgidum* subsp. *durum* (Avonlea, Canyon, Cappelli, Ciccio, Duilio, Latino, Simeto, Svevo) and two accessions of the ssp. *dicoccoides* (MG4328 and MG4413), were sub-chosen from a collection of 230 tetraploid wheat (*Triticum turgidum* L.) genotypes described by Marcotuli *et al*.^30^ and characterized by different total β-glucan content. A randomized complete block design with three replications and plots consisting of 1-m rows, 30 cm apart, with 80 germinating seeds per plot, was used in the field experiments. During the growing season, 10 g of nitrogen per m^2^ was applied at the beginning of planting and standard cultivation practices were adopted. Plots were hand harvested at maturity and grain was stored at 4 °C. Using the 1093 Cyclotec Sample Mill (Tecator Foss, 119 Hillerød, Denmark), the grain was ground and passed across a 1 micron sieve. Endosperm from each genotypes was collected in five developmental stages (6, 12, 21, 28 and 40 days after pollination) and stored at −80 °C for subsequent RNA extraction.

Mature kernels were used to determine the total β-glucan content by the Mixed-Linkage β-glucan Assay Kit (Megazyme International Ireland Ltd, Wicklow, Ireland) based on the accepted method by McCleary and Codd^35^ and included the industrial standard for barley (4.1% of β-glucan).

Analysis of variance, LSD and coefficient of variation were carried out for each trait using GenStat14 (version 18, VSN International Ltd, Hemel Hempstead, UK). Correlation analysis was conducted between *CslF6* and *CslH* genes expression and β-glucan content at different developmental stages.

### Cellulose synthase genes (*CslF6* and *CslH*) isolation and characterization

The sequences of cellulose synthase-like genes from *Oryza sativa* and *Hordeum vulgare* were downloaded from the Rice Genome Annotation Project database (http://rice.plantbiology.msu.edu/)^36^ and EnsemblePlants (http://plants.ensembl.org/Hordeum_vulgare/Info/Index)^37^, respectively, and used as initial query probe in the Durum Wheat Genome Database cv. Svevo (http://d-data.interomics.eu/node/1)^38^ for the wheat *Csl* sequences. Subsequently, the genomic and cDNA sequences were isolated and characterized amplifying target DNA from cv. Svevo. Structure of both *Csl*F6 and *CslH* genes was reported by all genome specie browsers and confirmed by FGENESH program (http://www.softberry.com/berry.phtmltopic=fgenesh&group=programs&subgroup=gfind)^39^.

The two *CslF6* and *CslH* whole sequences were blasted against the available dataset of SNP marker sequences reported by Wang *et al*.^40^, and SNPs with ≥80% identity were considered within the *Csl* genes.

### RNA extraction and cDNA synthesis

Annotated cDNA sequences from the two wheat genes *CslF6* (KP260638.1) and *CslH* (AK332242.1) were used for the primer pairs design. No differences were detected among A and B genomes for both genes, therefore no genome specific primer were designed. In addition, at this stage we were interested in understanding the expression profile of the two genes during the developmental stages and among different genotypes. A specific primer pair for *CslF6* was designed on exon 3 starting at 1.533 bp and with a sequence length of 260 bp, while *CslH* gene primers were picked at 420 bp of the last exon and counting of 195 bp sequence (Table 5).

**Table 5:** *Csl* primer combinations used for the qPCR experiments. For each primer forward and revers sequences and product length are reported.

Gene name	Forward sequence (5′-3′)	Reverse sequence (5′-3′)	Product length	Annealing temperature (°C)	Position
*TdCslF6*	AGTCGTGGACAGTGGACAGG	GGGGTCTTCTTCAACTTCTG	260	64	Exon 3
*TdCslH*	TGCTGTGGCTGGATGGTGTT	TTCTGCACAGAACCGAAATT	195	65	Exon 9

In order to analyse the expression level of the two genes, total RNA was extracted from the endosperm of each genotypes using the RNeasy Plant Mini Kit (QIAGEN®) and checked on 1.5% denaturing agarose gel. All RNA samples were lead to the same concentration (1 μg) and reverse-transcribed into double stranded cDNA with QuantiTect Reverse Trascriptase Kit (QIAGEN®). Data were normalized using three reference genes: Cell Division Control AAA-Superfamily of ATPases (*CDC*), ADP-Ribosilation Factor (*ADP-RF*) and RNase L Inhibitor-like protein (*RLI*)^41,42^. These genes have a stability value around 0.035 when evaluated with NormFinder software^43^.

### qPCR for cellulose synthase genes

Quantitative Real-Time PCR was carried out using EVA^®^ GREEN in the CFX96TM Real time PCR Systems (BIO-RAD). The PCR cycle was: 95 °C for 3 min followed by 40 cycles of 95 °C for 10 sec and at 60 °C for 30 sec. Amplification efficiency (98% to 100%) for the primer set was determined by amplification of cDNA with a series of six scalar dilution (1:5) per reaction. Each 10 µl PCR reaction contained 1 μl of a 1:5 dilution cDNA, 5 µl of EvaGreen Mix 10× (Bio-Rad), and 500 nM of each primer. Fluorescence signals were collected at each polymerization step. The specificity of the amplicons was confirmed by the presence of a single band of expected size for each primer pair in agarose gel (2% w/v), by a single peak melting curves of the PCR products and by sequencing of the amplified fragments (3500 Genetic Analyzer, Applied Biosystems). qRT-PCR data for both genes were derived from the mean values of three independent amplification reactions carried out on five different plants harvested in the same phenotypic stage (biological replicates). All calculations and analyses were performed using CFX Manager 2.1 software (Bio-Rad Laboratories) using the ΔC_t_ method, which used the relative quantity (RQ) calculated with a ratio of the RQ of the target gene to the relative expression of the reference gene (including the three reference targets in each sample). Standard deviations were used to normalize values for the highest or lowest individual expression levels (CFX Manager 2.1 software user manual, Bio-Rad Laboratories). The ANOVA and LSD test were used to underline significant differences between the genotypes for the two considered *Csl* genes.

## Conclusion

Durum wheat kernel contains macronutrients such as protein, fat, and carbohydrate that are required by humans for growth and maintenance, and also important minerals, vitamins, and other micronutrients essential for optimal health. It has been demonstrated that whole grain cereal foods have a key role on improving human health and lower the risk of serious, diet-related diseases. Dietary fibre is one of the most important components of whole grain cereals from this standpoint. In particular, β-glucan make up an important proportion of dietary fibre in many diets.

The identification of genes involved in the biosynthesis of the β-glucan opened the way for the genetic improvement of cereal quality parameters important in human health. Different genes are involved in β-glucan biosynthesis of different tissues/cell types, with *CslF6* and *CslH* genes making the main part of the process.

The results presented here represent additional information on the gene sequences for gene involved in β-glucan pathway, biosynthesis and accumulation of β-glucan in durum wheat. The data not only are consistent with what reported in barley, but also contribute to our understanding of the genetic complexity of this important agronomic trait. It allowed us the evaluation of the *CslF6* and *CslH* transcript variation in wheat endosperm at different developmental stages (from 6 to 40 dap). In addition, the correlation analysis between the final β-glucan amount in wheat grains and the expression levels of these two genes open a way for further genetic studies on other genes involved in the biosynthesis and degradation of β-glucan.

## Electronic supplementary material

Supplementary Dataset 1
