# Author Correction: Expression analysis of cellulose synthase-like genes in durum wheat

**DOI:** 10.1038/s41598-020-57688-2

**Published:** 2020-01-22

**Authors:** Ilaria Marcotuli, Pasqualina Colasuonno, Antonio Blanco, Agata Gadaleta

**Affiliations:** 1 Department of Agricultural and Environmental Science, University of Bari ‘Aldo Moro’, Via G. Amendola 165/A, 70126 Bari, Italy; 2 Department of Soil, Plant and Food Sciences, University of Bari ‘Aldo Moro’, Via G. Amendola 165/A, Bari, Italy

Correction to: *Scientific Reports* 10.1038/s41598-018-34013-6, published online 23 October 2018

The original version of this Article contained errors in the names of the authors Ilaria Marcotuli, Pasqualina Colasuonno, Antonio Blanco & Agata Gadaleta which were incorrectly given as Marcotuli Ilaria, Colasuonno Pasqualina, Blanco Antonio & Gadaleta Agata respectively.

These errors have now been corrected in the PDF and HTML versions of the Article, and in the Supplementary Dataset file.

