# Supplementary material for: Expression analysis of cellulose synthase-like genes in durum wheat
Source: Sci Rep. 2018 Oct 23;8:15675. doi: 10.1038/s41598-018-34013-6 (PMC6199314; doi:10.1038/s41598-018-34013-6)
Supplement: Supplementary file 1 — Supplementary Dataset 1 [file 41598_2018_34013_MOESM1_ESM.docx]

**Expression analysis of cellulose synthase-like genes in durum wheat**

Ilaria Marcotuli^1^, Pasqualina Colasuonno^1^*, Antonio Blanco^2^, Agata Gadaleta^1^*

^1^Department of Agricultural and Environmental Science, University of Bari ‘Aldo Moro’, Via G. Amendola 165/A, 70126, Bari, Italy

^2^Department of Soil, Plant and Food Sciences, University of Bari 'Aldo Moro', Via G. Amendola 165/A, Bari, Italy

Corresponding authors [pasqualina.colasuonno@uniba.it](mailto:pasqualina.colasuonno@uniba.it) ; agata.gadaleta@uniba.it

**>CslF6_Svevo_7A_genomic DNA**

ATGGCGCCAGCGGTGGCCGGAGGAGGCCGCGTGCGGAGCAATGAGCCGGCTGCTGCCGCGGCGCCGGCGGCCGCCAGCGGGAAGCCCTGCGTCTGCGGCTTCCAGGTGTGCGCCTGCACGGGATCGGCCGCGGTGGCGTCCGCCGCCTCCTCGCTGGACATGGACATCGTGGCCATGGGCCAGATCGGCGCCGTCAACGACGAGAGCTGGGTGGGCGTGGAGCTCGGCGAAGACGGCGAGACCGACGAAAGCGGTGTCGCCGTTGACGACCGCCCCGTCTTCCGCACCGAGAAAATCAAGGGTGTCCTTCTCCACCCCTACCGGTACGTGCAACTAAGAGCATCCTAGTGGCTTGTCTAAATTTAATAGTCTATATCTACATATACACAATGGTGTAAAAAGATTTTCTTTTAGACAACCTAAGTTTTCCAGCGGATTGTCTATATATATGATGTCTATATTCTCTTTCATATATTTTAATAGTATCTTATAGCAGCTATATTTTCTAAAAATCTTATGTTAGTCATTTCTGAGTTGTTTTCATTTATTTTTTTCATCCGCAAGACATATAAAGGAGAAAATAAACCAGCCGCGAAATTATTTTAGGTTCAACACTAGCAACACCCTATATAATTTACGAGAAATAAACAATAGCAACAGGTTCAACCCTGATGAGGCGACGGTCCAGGCGCAACGACCTCCGTGTCCAGCGCCGGCGCGGCCGCCTCCCTGCCGACGTCCTCCAATTGCTCCGAATTGGTGAATGGCTGAGCTCATGAGCCAACATTTTTGGTTTAGACATTGTGGCTTGGTTGTCCAAATGTGGACGACGGGTAGTTAGTTTGGACATTGTCTAAATTTTAGATGACGCAATAGACAAGTCACTGAAGACGTTTTTTGACATGTGGTAGTCTAAATTTGGTATAGACCACCATTTGCACAAGCCACTAAAAATGCTCTAAACTCCCTATATCTGCGTCACAGTAGTAACAATTACTAGTAGTATTTCATACTTATTTACTGTGTAAGTATTTATTATTACTATTATTTTGCACAACAAGTCTAAAATGATTAAGCCAACAAGCACCACGCCAGGCTGACAGTCAGGATTATTTGATCTTGACCGGTGGCGGTGCTCATCGAGACTGGTACTAGTCCTAGGACTGGTAGCGTGGTCTGGTCGGTGTTAATCACAGTAATTAATAGGTAATTAATGTAAACATGGATTAAAATTTGACAAGTCCGAGACAGGTGCACGTTAGAGGCGGGCCAATGATGGCTCGAATCCACTCAAAACAGCGCGTCCCGGTGTGGGCTGTCGGCTCGGTGCGGCGGGGCGCTGGTTCTTCCATCTTACTACTAGTCATAGTCACTGCTGCATGGGCCCGCTTAAGGGGACGTTAGCCGTTGGGCCTGCCTGGCAGGTGGGCCCCGGTGGCCACCCTGGCGGCTCATAAATCCTTGCCACTTTAGAGTTAATAAAATCTGGGTGTGCGGGTGGGATGGATTCGGTTGCAATGGCAATAGGAATCCGAGGTGAAAGGAGATGAGATGACAGTGGGCATGGCTTGCACGTGAATCCAAGCCACATCATTAAAAGCCTCGTCGCCATCCTCCCTGGGGACGTCGCAGTGAGAAAGTTAGTTAAACTTTTTGGGTTGGGACAAGGTATAAATAAAAGTAAGTAACATGTCCTTTTTTGTCACTGGAGAAACGTATGTATGTATGGTATGGTAGTATATGAGCATCTAGTCTAGTTATAGATGGATGATGCTACCGCTGGAGCCTCTAGTACGTTAGTTTTTCTTCAGCCGTTTTATGGAGAAAACGAGAAGAAGATTATCATGTCATGTCATGTCATAGAAAGGAGGAGAAGCAAAAAAAAAGGTGGTGCCATTGCCACCGCTTGAATGCCTTTTTTCAGAAGAATGCGTGAGTCATGTTGGCACCGAGAAAAGCCATATTAAAGTGGCAGGGTTACAACTCAGAAGAAATGCGCGTGCTAGATAAACACTAGGATATGTGAAGTGCACTCGGCACAACATCTTCAAAGTACTCCAAAGATAAATAACAAGAAAAAGATTACTACTACTCCAAAGTACTCCTACTACTACTAGTAACGATTAGCAGGAGAAGGTTCCAACGACTTTTTGGCGCCAATAGCATAAAGAAGAAGAAGAAGAAGAAAATAACGAGAAAAGAGGCCCATTAATGGAGCAACGAATCCAGCGGCACCACCTCTGGCGGTCGCGTCCATGCCCTCGCACGACGGGTGAGGGAGGGAGGGGCCCGGGCCTACTGACAGCCGAGGCATGTCGGTGCTCATACACGGCGCCGTTTGCTGCCAAGTGTGCCAGCTCACACTCATTGACTTGCCAGCTCCCGCCTTGGCTGTCAATGAGAACATGATGCCCTTCTCTCTCTCTCTCGCCTTTTGGCATTTGCAAAAAATTAAAACTAGCTGTCTGATAGGGAAAAGAAATGCAAAGGGAAAAGATGAACGTGGCGCATGTTCCCTCCAATAATTGCACCCAATCATCACTCGATTAATCCAACAATTTTTACTTTACTACTCCCACAAGAACTGATGATGAACTGTAACTGACGGTGAATGTGAATAATGCAATGCAGGGTGCTGATCTTCGTTCGGCTGATCGCCTTCACCCTGTTCGTGATCTGGCGTATCTCCCACAAGAACCCTGACGCCATGTGGCTGTGGGTGACATCCATCTGCGGCGAGTTCTGGTTCGGCTTCTCCTGGCTGCTGGATCAGCTGCCCAAGCTGAACCCCATCAACCGCGTGCCGGACCTGGCCGTGCTGCGGCAGCGCTTCGACCGCCCCGACGGCACCTCCACCCTCCCGGGGCTCGACATCTTCGTCACCACGGCCGACCCCATCAAGGAGCCCATCCTCTCCACCGCCAACTCCGTGCTCTCCATCCTCGCCGCCGACTACCCCGTCGACCGCAACACATGCTACGTCTCTGACGACAGTGGCATGCTGCTCACCTACGAGGCCCTGGCCGAGTCCTCCAAGTTCGCCACCCTCTGGGTGCCCTTCTGCCGCAAGCACGGGATCGAGCCCAGGGGTCCGGAGAGCTACTTCGAGCTCAAGTCCCACCCCTACATGGGGAGAGCCCAGGACGAATTCGTCAACGACCGCCGCCGCGTCCGCAAGGAGTACGACGAGTTCAAGGCCAGAATCAACAGCCTGGAGCACGACATCAAGCAGCGCAACGACGGGTACAACGCCGCCAACGCCCACCGGGAAGGCGAGCCCCGACCCACCTGGATGGCCGACGGCACCCAGTGGCAGGGCACCTGGGTGGACGCCTCCGAGAACCACCGCAGGGGCGACCACGCCGGCATCGTCCTGGTCAGTACTAGTTGCTATACTTATCACTAGGACTACCATTTACTTAGGGTCTCTTTCGTCCGTGCATGATGCATGCATGCTGCTGTTCTTGGAATCTTGGTTAGTTAGGGCCTCGTTATTAGTGGCCATCTGATGTGATGCCTGCCTGCACTGCACTGCTGTGCCGATCCAAGGGAGATTTTGACAGAATGGACGTGGTGATGGTCGAGAGTGCAACCACCGGCCGGCCAGCCAAGCACACTTGTGGACAGACATGGAAGAAAACATGCATGCTTCTCTTCTCGCCTCGTCCTGTGGCCAGCAACGTGTGGTTCCACTCACTCACGCTGTGACGAGGAATGGTGGTTGGGGTGGTCCTTTCCCCCCGACAGCACTACAGCGTCCACTTTATGACCCATTTAATTCAATTCACCGGCCCTGCTTTCTTAACTGCCTCAATCATTCATCAGTTTGTTTACTCTGCCCAACTCTTAACCCTGTACTGTAGTATACTTAGTAGTACTAATAATTAATTACTCCAACCACTAATCACCTTAAGGTAGTAATAGTAACACTCCAGTAAGAATCATTTGACCTTTTACTATGGCGATCGAGAAGTAAAAGTAACAGCTAAAATTAACCGTGTCATTCATTTAACCTTGTTTTTTTTTACCACTATCTACCTAAGCTCAAGTTGTGATTGTACTGCAAGAGGAATGGAGTGCTGACAATGGTGTGTGTGCAATGGATGCAGGTGCTGCTGAACCACCCGAGCCACCGCCGGCAGACGGGCCCGCCGGCGAGCGCTGACAACCCACTGGACTTCAGCGGCGTGGATGTGCGTCTCCCCATGCTGGTGTACGTGTCCCGTGAGAAGCGCCCCGGACACGACCACCAGAAGAAGGCCGGCGCCATGAACGCGCTCACCCGCGCCTCCGCGCTGCTCTCCAACTCCCCCTTCATCCTCAACCTCGACTGCGACCATTACATCAACAACTCCCAGGCCCTCCGCGCCGGCATCTGCTTCATGGTGGGACGCGACAGCGACACCGTCGCCTTCGTCCAGTTCCCGCAGCGCTTCGAGGGCGTCGACCCCACCGACCTCTACGCCAACCACAACCGCATCTTCTTCGACGGCACCCTCCGTGCCCTCGACGGCATGCAGGGCCCCATCTACGTCGGCACCGGCTGTCTCTTCCGCCGCATCACCGTCTACGGCTTCGACCCGCCCAGGATCAACGTCGGCGGGCCCTGCTTCCCCAGGCTCGCCGGGCTCTTCGCCAAGACCAAGTACGAGAAGCCCGGCCTCGAGATGACCATGGCCAAGGCCAAGGCCGCGCCGGTGCCCGCCAAGGGCAAGCACGGCTTCCTGCCTCTGCCCAAGAAGACGTACGGCAAGTCGGACGCCTTCGTGGACAGCATCCCGCGCGCGTCGCACCCGTCGCCTTACGCCGCGGCGGCTGAGGGCATCGTGGCCGACGAGGCGACCATCGTGGAGGCGGTGAACGTGACGGCCGCGGCGTTCGAGAAGAAGACCGGCTGGGGCAAAGAGATCGGCTGGGTGTACGACACCGTGACGGAGGACGTGGTGACCGGGTACCGGATGCATATCAAGGGGTGGCGGTCACGCTACTGCTCCATCTACCCACACGCCTTCATCGGCACCGCACCCATCAACCTCACGGAGAGGCTCTTCCAGGTGCTCCGCTGGTCCACGGGCTCCCTCGAGATCTTCTTCTCCAAGAACAACCCGCTCTTCGGCAGCACCTACCTCCACCCGCTGCAGCGCGTCGCCTACATCAACATCACCACCTACCCCTTCACCGCCATCTTCCTCATCTTCTACACCACCGTGCCGGCGCTCTCCTTCGTCACCGGCCACTTCATCGTGCAACGCCCCACCACCATGTTCTACGTCTACCTGGGCATCGTGCTCTCCACGCTGCTCGTCATCGCCGTGCTGGAGGTCAAGTGGGCCGGGGTCACCGTCTTCGAGTGGTTCAGGAACGGCCAGTTCTGGATGACGGCAAGTTGCTCCGCCTACCTCGCCGCCGTGTGCCAGGTGCTGACCAAGGTGATATTCCGGCGTGACATCTCCTTCAAGCTCACATCCAAGCTACCGTCCGGAGACGAGAAGAAGGACCCCTACGCCGACCTGTACGTGGTGCGCTGGACGCCGCTCATGATCACACCCATCATCATCATTTTCGTCAACATCATTGGGTCGGCGGTGGCCTTCGCCAAGGTGCTCGACGGCGAGTGGACGCACTGGCTCAAGGTCGCCGGCGGGGTCTTCTTCAACTTCTGGGTGCTGTTCCACCTCTACCCGTTCGCCAAGGGGATCCTGGGGAAGCACGGCAAGACGCCAGTCGTGGTGCTCGTCTGGTGGGCATTCACCTTCGTCATCACCGCCGTGCTCTACATCAACATCCCCCACATGCATAGCTCGGGAGGCAAGCACACAACGGTGCATGGTCACCATGGCAAGAAGTTCGTCGACGCAGGGTACTACAACTGGCCATGAGGTCCCTGTTGACGACTTTGCCGCCGGACAGGACGACCTGAGACAAGAAGCAACAAGTCATCCACTGAACAGTGCA

**>CslF6_7A_CDS**

ATGGCGCCAGCGGTGGCCGGAGGAGGCCGCGTGCGGAGCAATGAGCCGGCTGCTGCCGCGGCGCCGGCGGCCGCCAGCGGGAAGCCCTGCGTCTGCGGCTTCCAGGTGTGCGCCTGCACGGGATCGGCCGCGGTGGCGTCCGCCGCCTCCTCGCTGGACATGGACATCGTGGCCATGGGCCAGATCGGCGCCGTCAACGACGAGAGCTGGGTGGGCGTGGAGCTCGGCGAAGACGGCGAGACCGACGAAAGCGGTGTCGCCGTTGACGACCGCCCCGTCTTCCGCACCGAGAAAATCAAGGGTGTCCTTCTCCACCCCTACCGGGTGCTGATCTTCGTTCGGCTGATCGCCTTCACCCTGTTCGTGATCTGGCGTATCTCCCACAAGAACCCTGACGCCATGTGGCTGTGGGTGACATCCATCTGCGGCGAGTTCTGGTTCGGCTTCTCCTGGCTGCTGGATCAGCTGCCCAAGCTGAACCCCATCAACCGCGTGCCGGACCTGGCCGTGCTGCGGCAGCGCTTCGACCGCCCCGACGGCACCTCCACCCTCCCGGGGCTCGACATCTTCGTCACCACGGCCGACCCCATCAAGGAGCCCATCCTCTCCACCGCCAACTCCGTGCTCTCCATCCTCGCCGCCGACTACCCCGTCGACCGCAACACATGCTACGTCTCTGACGACAGTGGCATGCTGCTCACCTACGAGGCCCTGGCCGAGTCCTCCAAGTTCGCCACCCTCTGGGTGCCCTTCTGCCGCAAGCACGGGATCGAGCCCAGGGGTCCGGAGAGCTACTTCGAGCTCAAGTCCCACCCCTACATGGGGAGAGCCCAGGACGAATTCGTCAACGACCGCCGCCGCGTCCGCAAGGAGTACGACGAGTTCAAGGCCAGAATCAACAGCCTGGAGCACGACATCAAGCAGCGCAACGACGGGTACAACGCCGCCAACGCCCACCGGGAAGGCGAGCCCCGACCCACCTGGATGGCCGACGGCACCCAGTGGCAGGGCACCTGGGTGGACGCCTCCGAGAACCACCGCAGGGGCGACCACGCCGGCATCGTCCTGGTGCTGCTGAACCACCCGAGCCACCGCCGGCAGACGGGCCCGCCGGCGAGCGCTGACAACCCACTGGACTTCAGCGGCGTGGATGTGCGTCTCCCCATGCTGGTGTACGTGTCCCGTGAGAAGCGCCCCGGACACGACCACCAGAAGAAGGCCGGCGCCATGAACGCGCTCACCCGCGCCTCCGCGCTGCTCTCCAACTCCCCCTTCATCCTCAACCTCGACTGCGACCATTACATCAACAACTCCCAGGCCCTCCGCGCCGGCATCTGCTTCATGGTGGGACGCGACAGCGACACCGTCGCCTTCGTCCAGTTCCCGCAGCGCTTCGAGGGCGTCGACCCCACCGACCTCTACGCCAACCACAACCGCATCTTCTTCGACGGCACCCTCCGTGCCCTCGACGGCATGCAGGGCCCCATCTACGTCGGCACCGGCTGTCTCTTCCGCCGCATCACCGTCTACGGCTTCGACCCGCCCAGGATCAACGTCGGCGGGCCCTGCTTCCCCAGGCTCGCCGGGCTCTTCGCCAAGACCAAGTACGAGAAGCCCGGCCTCGAGATGACCATGGCCAAGGCCAAGGCCGCGCCGGTGCCCGCCAAGGGCAAGCACGGCTTCCTGCCTCTGCCCAAGAAGACGTACGGCAAGTCGGACGCCTTCGTGGACAGCATCCCGCGCGCGTCGCACCCGTCGCCTTACGCCGCGGCGGCTGAGGGCATCGTGGCCGACGAGGCGACCATCGTGGAGGCGGTGAACGTGACGGCCGCGGCGTTCGAGAAGAAGACCGGCTGGGGCAAAGAGATCGGCTGGGTGTACGACACCGTGACGGAGGACGTGGTGACCGGGTACCGGATGCATATCAAGGGGTGGCGGTCACGCTACTGCTCCATCTACCCACACGCCTTCATCGGCACCGCACCCATCAACCTCACGGAGAGGCTCTTCCAGGTGCTCCGCTGGTCCACGGGCTCCCTCGAGATCTTCTTCTCCAAGAACAACCCGCTCTTCGGCAGCACCTACCTCCACCCGCTGCAGCGCGTCGCCTACATCAACATCACCACCTACCCCTTCACCGCCATCTTCCTCATCTTCTACACCACCGTGCCGGCGCTCTCCTTCGTCACCGGCCACTTCATCGTGCAACGCCCCACCACCATGTTCTACGTCTACCTGGGCATCGTGCTCTCCACGCTGCTCGTCATCGCCGTGCTGGAGGTCAAGTGGGCCGGGGTCACCGTCTTCGAGTGGTTCAGGAACGGCCAGTTCTGGATGACGGCAAGTTGCTCCGCCTACCTCGCCGCCGTGTGCCAGGTGCTGACCAAGGTGATATTCCGGCGTGACATCTCCTTCAAGCTCACATCCAAGCTACCGTCCGGAGACGAGAAGAAGGACCCCTACGCCGACCTGTACGTGGTGCGCTGGACGCCGCTCATGATCACACCCATCATCATCATTTTCGTCAACATCATTGGGTCGGCGGTGGCCTTCGCCAAGGTGCTCGACGGCGAGTGGACGCACTGGCTCAAGGTCGCCGGCGGGGTCTTCTTCAACTTCTGGGTGCTGTTCCACCTCTACCCGTTCGCCAAGGGGATCCTGGGGAAGCACGGCAAGACGCCAGTCGTGGTGCTCGTCTGGTGGGCATTCACCTTCGTCATCACCGCCGTGCTCTACATCAACATCCCCCACATGCATAGCTCGGGAGGCAAGCACACAACGGTGCATGGTCACCATGGCAAGAAGTTCGTCGACGCAGGGTACTACAACTGGCCATGA

**>CslF6_Svevo_7B_genomic**

ATGGCGCCAGCGGTGGCCGGAGGAGGCCGCGTGCGGAGCAATGAGCCTGCTGCTGCCGCTGCCAGCGACAAGCCCTGCGTGTGCGGCTTCCAGGTGTGCGCCTGCACGGGATCGGCCGCGGTGGCGTCCGCCGCCTCCTCGCTGGACATGGACATCGTGGCCATGGGGCAGATCGGCGCCGTCAACGACGAGAGCTGGGTGGGCGTGGAGCTCGGCGAGGACGGCGAGACCGACGAAAGCGGTGCCGCCGTTGACGACCGCCCCGTCTTCCGCACCGAGAAAATCAAGGGTGTCCTCCTCCACCCCTACCGGTACGTGCAACAAAACTCCCTATATCTGCGTCATAGTAACAATTACTACTTACTATTTCATACTTACTGTAAGAAGTATTTATTTATTATTACTCTTTTGCACAACAAGTCTAAAATGATTAAACCAACAAGCACCACGCCAAGGTTGACAGTCAGGATTATTTGATCTTGACCGGTGGCGGTGCTCATGAAGATTACTAGTATCGCTAGTACTCCTAGGACTGCTACTAGTAGTAGCATTGTCTGGTCGGTGTTAATCACAGTAAAAAATAGTTAATTAATGTAAACCTGGATTAAAAATTGATAAGTCCGAGACAGGTCACGTTAGAGGCGGGCCAATGATGGCTCGAATCCACCCAAAACAGCGCGTCCCGGTGTGGGCTGTCGGCTCGGTGCGGCGGGGCGCTGGTTCTTCCATTTTATTACTACTAGTCATAGTCACTGCTGCATGGGCCCGCGGGAGGGGACGTTAGCCGTTGGGCCTGCCTGGCAGGTGGGCCCCGGTGGCCACCCTGGCGGCTCATAAATCCTTGCCACTTTAGATAGTAGAGTTAATTAATAAAATGTGGGTGTGCGGGGGGGTTGGCCTCTCTGCAATAGCAATAGGAATCCGAGGTGAAACGAGATGACAGTGGGCTTGGCTTGCACGTGAATCCAAGCCACATCATTAAAAGCCTCGTCGCCATTCTCCCTGGGGACGTCGCAGTGAGAAAGTTGGTTAAACTTTTAGGGTTCGGACAAGATATAAAACTAAGTAACATGTCCTTTTTTGTCACTGGAGAAACGTAGTATGGTATGGTAGTATATGAGATTTTTCGGCATCTAGTTATAGATGGATGATGCTACTGCTGGAGCCTGTAGTACGTTAGTTTTTGTTCAGCCGTTTTATGGGGAGAAAACGAGAAGAAGATTATCATGTCATAAAAAGGAGGAGAAGCAAAAGAGGTGGTGCCACCGCATGAATGCCTTTTTCCCCAGAAGAATGCGTGAGTCATGTTGGCACCGAGAAAAGCCATATTAAAGTGGCAGAGTTACAACTCAGAAGAAATGCGCGTGTCCCCTCAAAGAAGAAGAAGAAGAAATGCGCGTGCTAGATAAACACTACTACTAGGATATGTGAAGTGCACTCGGCACAACATCTTCAAAGCACTCCAAAGATTAACAAGAAAAAGATTACTCCTACTCCAAAGTACTCCTACTAGTGGTAGTAACGATTAGCAGGAGAAGGTTCCAACGACTTTTTGGCGCCAATAGCACAAATAAAGAAGAAGAAAAAAACTTACTAACGAGAAAAGAGGCCCATTAATGGAGCAAGGAATCCAGCGGCACCACCTCTGGCCCGCGCACGACGGACGGGTGAGGGAGGGGCCCGGGCCTACTGACAGCCGAGGCATGTCGGTGCTCATACACGGCGCCGTTTGCTGCCAAGTGTGCCAGCTCACACTCATTGACTTGCCAGCTCCCGCCTTGGCTGTCAATGAGAACATGATACCGTTCTCTCTCTTTCTCTCTCTCGCCTTTTGGCATTTGCAAAAGAAATTAGAACTAGCTGTCTGATAGGGAAAAGAAATGCAAAGGGCGCATGTTCCCTCCAATAATTGCACCCAATCATCGTACCAACATACTCGATTAATCCAAACATTTTTTTACTAGTAAGAGTGAGTTGATGATGAAGTGTAACTGACGGTGAATGTGAATAATGCAATGCAGGGTGCTGATCTTCGTCCGTCTCATCGCGTTCACCCTGTTCGTGATCTGGCGTATCTCCCACAAGAACCCTGACGCCATGTGGCTGTGGGTGACATCCATCTGCGGCGAGTTCTGGTTCGGCTTCTCCTGGCTGCTGGATCAGCTCCCCAAGCTGAACCCCATCAACCGCGTGCCGGACCTGGCCGTGCTCCGGCAGCGCTTCGACCGCCCCGACGGCACCTCCACGCTCCCTGGGCTGGACATCTTCGTCACCACGGCCGACCCCATCAAGGAGCCCATCCTCTCCACCGCCAACTCGGTGCTCTCCATCCTCGCCGCCGACTACCCCGTGGACCGCAACACATGCTACGTCTCCGACGACAGTGGCATGCTGCTCACCTACGAGGCCCTGGCTGAGTCCTCCAAGTTCGCCACCCTCTGGGTGCCCTTCTGCCGCAAGCACGGGATTGAGCCGAGGGGCCCGGAGAGCTACTTCGAGCTCAAGTCCCACCCCTACATGGGGAGAGCCCAGGACGAGTTCGTGAACGACCGCCGACGCGTCCGCAAGGAGTACGACGAGTTCAAGGCCAGGATCAACAGCCTGGAGCATGACATCAAGCAGCGCAACGACGGGTACAACGCCGCCAACGCTCACCGGGAAGGCGAGCCCCGACCCACCTGGATGGCCGACGGCACCCAGTGGGAGGGCACCTGGGTCGACGCCTCCGAGAACCACCGCAGGGGCGACCACGCCGGCATCGTCCGGGTCAGTACTTACTATTCTTATTATCACTACCATTTACTTACTCCTACTTACTGTCTCTTTCCTACTAGAGTACATGTCTGCATGCATGCATGCTGCTGTTGTTGGAATCTTGGTTAGTAGTTAGGGCCTCGTTATTAGGGCATGTACAATGCATAGCCTTAAGGTGATGCCTCGCATGCCATGTAGGATCGGATATGACGTAAAGTAGGTTCGGATAGAGAAGCGGGATCCTCTCCAGGAGGCGGGTGCTTGAAGAGAAAATATGTGGTCCGGTGACAAAAGCTGAAAAGGTTGGAGTGAAAAATAAAGATGCATGTATACTAGAGTCTTTATTTTTTATTTTTTAATGAGGCCCACTAGTGATAGCTTGCATTGAAGAGAAAAAATTAATGTAGATGCTTTAAATTAGTTTTTGTCATGAGGCATATGTATTCATATGCCACCATTGTACATGCCCTTAGTGGCCATCTGATGTGATGCCTGCCTGCACTGCTGTGCCGATCCAAGGGAGATTTTGACAGAATGGACGTGGTGATAGTCGAGAGTGCAACCACCGGCCGGCCAGCCAAGCACACTTGTGGACAGACATGGAAGAGAACATGCATGCTTCTCTTCTCGCATCCGTCCTGTGGCCACCAACGTGTGGTTCCACTCATTCATGCTGTGACGAGGAATGGTGGTTGGGGTGGTCCTTTCCCCCCGACACCACTACAGCCTCCACTTTATGACCCATTTAATTCAATTCACCGGCCCTGCTTCTTTGTTAACTGCCTCAATCATTCATCACTTTACTCTGCCCAACTCTTAACCCTGTCTGTAGTAATAATTAATTACTCCAACCACTAATCACCCTAAGGTACCCCATAGTAGTAGTAACACTCCAGTAAGAATCATTTGACCTTTTATTACTATGGAGCAGTAACAGCTAAAATTAACCATGTCATTCATTTAACCTTGTTTTTTTTACTACCACTATCTGCCTAAGCTCAAGTTGTGATTTATCCGCAAAAAAGAAAAAGAGTCAAGTTGTGATTGTACTACTAGTGCAAGAGGAATGGAGTGCTGACAATGGTGTGTGTGCAATGGATGCAGGTGCTGCTGAACCACCCGAGCCACCGGCGGCAGACGGGCCCGCCGGCGAGCGCTGACAACCCACTGGACTTCAGCGGCGTGGATGCGCGTCTCCCCATGCTGGTGTACGTTTCCCGTGAGAAGCGCCCCGGACACGACCACCAGAAGAAGGCCGGCGCCATGAACGCGCTCACCCGCGCCTCCGCGCTGCTCTCCAACTCCCCCTTCATCCTCAACCTCGACTGCGACCATTACATCAACAACTCCCAGGCCCTCCGCGCCGGCATCTGCTTCATGGTGGGACGCGACAGCGACACCGTCGCCTTCGTCCAGTTCCCGCAGCGCTTCGAGGGCGTCGACCCCACCGACCTCTACGCCAACCACAACCGCATCTTCTTCGACGGCACCCTCCGTGCCCTCGACGGCATGCAGGGCCCCATCTACGTCGGCACCGGGTGTCTCTTCCGCCGCATCACCGTCTACGGCTTCGACCCGCCCAGGATCAACGTCGGCGGGCCCTGCTTCCCCAGGCTCGCCGGGCTCTTCGCCAAGACCAAGTACGAGAAGCCGAGCCTCGAGATGACAATGGCCAAGGCCAAGGCCGCGCCGGTGCCCGCCAAGGGGAAGCACGGCTTCCTGCCTCTGCCCAAGAAGACGTACGGCAAGTCGGACGCCTTCGTGGACAGCATCCCGCGCGCGTCGCACCCGTCGCCTTACGCGGCGGCGGCTGAGGGCATCGTGGCCGACGAGGCGACCATCGTGGAGGCGGTGAACGTGACGGCGGCGGCGTTCGAGAAGAAGACCGGGTGGGGCAAAGAGATCGGGTGGGTGTACGACACGGTGACGGAGGACGTGGTCACCGGGTACCGGATGCATATCAAGGGGTGGCGGTCACGCTACTGCTCCATCTACCCACACGCCTTCATCGGCACCGCACCCATCAACCTCACGGAGAGGCTCTTCCAGGTGCTCCGCTGGTCCACGGGCTCCCTCGAGATCTTCTTCTCCAAGAACAACCCGCTCTTCGGCAGCACCTACCTCCACCCGCTGCAGCGCGTCGCCTACATCAACATCACCACCTACCCCTTCACCGCCATCTTCCTCATCTTCTACACCACCGTGCCGGCGCTCTCCTTCGTCACCGGCCACTTCATCGTGCAGCGCCCCACCACCATGTTCTACGTCTACCTGGGCATCGTGCTCTCCACGCTGCTCGTCATCGCCGTGCTGGAGGTCAAGTGGGCCGGGGTCACCGTCTTCGAGTGGTTCAGGAACGGCCAGTTCTGGATGACGGCAAGTTGCTCCGCCTACCTCGCCGCCGTGTGCCAGGTGCTTACCAAGGTGATATTCCGGCGTGACATCTCCTTCAAGCTCACATCCAAGCTACCGTCGGGAGACGAGAAGAAGGACCCCTACGCCGACCTCTACGTGGTCCGCTGGACGCCGCTGATGATCACACCCATCATCATCATTTTCGTCAACATCATCGGATCCGCGGTGGCCTTCGCCAAGGTGCTGGACGGCGAGTGGACGCACTGGCTCAAGGTCGCCGGCGGGGTCTTCTTCAACTTCTGGGTGCTGTTCCACCTCTACCCGTTCGCCAAGGGCATCCTGGGGAAGCACGGCAAGACGCCAGTCGTGGTGCTCGTCTGGTGGGCATTCACCTTCGTCATCACCGCCGTGCTCTACATCAACATCCCCCACATGCATAGCTCGGGAGGCAAGCACACAACGGTGCATGGTCACCATGGCAAGAAGTTCGTCGACGCAGGGTACTACAACTGGCCGTGAGGTCCCTGTCCACTGTCCACGACTTTGCCGCCGCGCATCCGGACAAGATGACCTGAGACAAGAAACAACAAGTCATCCACTCAACAGTGCA

**>CslF6_Svevo_7B_CDS**

ATGGCGCCAGCGGTGGCCGGAGGAGGCCGCGTGCGGAGCAATGAGCCTGCTGCTGCCGCTGCCAGCGACAAGCCCTGCGTGTGCGGCTTCCAGGTGTGCGCCTGCACGGGATCGGCCGCGGTGGCGTCCGCCGCCTCCTCGCTGGACATGGACATCGTGGCCATGGGGCAGATCGGCGCCGTCAACGACGAGAGCTGGGTGGGCGTGGAGCTCGGCGAGGACGGCGAGACCGACGAAAGCGGTGCCGCCGTTGACGACCGCCCCGTCTTCCGCACCGAGAAAATCAAGGGTGTCCTCCTCCACCCCTACCGGGTGCTGATCTTCGTCCGTCTCATCGCGTTCACCCTGTTCGTGATCTGGCGTATCTCCCACAAGAACCCTGACGCCATGTGGCTGTGGGTGACATCCATCTGCGGCGAGTTCTGGTTCGGCTTCTCCTGGCTGCTGGATCAGCTCCCCAAGCTGAACCCCATCAACCGCGTGCCGGACCTGGCCGTGCTCCGGCAGCGCTTCGACCGCCCCGACGGCACCTCCACGCTCCCTGGGCTGGACATCTTCGTCACCACGGCCGACCCCATCAAGGAGCCCATCCTCTCCACCGCCAACTCGGTGCTCTCCATCCTCGCCGCCGACTACCCCGTGGACCGCAACACATGCTACGTCTCCGACGACAGTGGCATGCTGCTCACCTACGAGGCCCTGGCTGAGTCCTCCAAGTTCGCCACCCTCTGGGTGCCCTTCTGCCGCAAGCACGGGATTGAGCCGAGGGGCCCGGAGAGCTACTTCGAGCTCAAGTCCCACCCCTACATGGGGAGAGCCCAGGACGAGTTCGTGAACGACCGCCGACGCGTCCGCAAGGAGTACGACGAGTTCAAGGCCAGGATCAACAGCCTGGAGCATGACATCAAGCAGCGCAACGACGGGTACAACGCCGCCAACGCTCACCGGGAAGGCGAGCCCCGACCCACCTGGATGGCCGACGGCACCCAGTGGGAGGGCACCTGGGTCGACGCCTCCGAGAACCACCGCAGGGGCGACCACGCCGGCATCGTCCGGGTGCTGCTGAACCACCCGAGCCACCGGCGGCAGACGGGCCCGCCGGCGAGCGCTGACAACCCACTGGACTTCAGCGGCGTGGATGCGCGTCTCCCCATGCTGGTGTACGTTTCCCGTGAGAAGCGCCCCGGACACGACCACCAGAAGAAGGCCGGCGCCATGAACGCGCTCACCCGCGCCTCCGCGCTGCTCTCCAACTCCCCCTTCATCCTCAACCTCGACTGCGACCATTACATCAACAACTCCCAGGCCCTCCGCGCCGGCATCTGCTTCATGGTGGGACGCGACAGCGACACCGTCGCCTTCGTCCAGTTCCCGCAGCGCTTCGAGGGCGTCGACCCCACCGACCTCTACGCCAACCACAACCGCATCTTCTTCGACGGCACCCTCCGTGCCCTCGACGGCATGCAGGGCCCCATCTACGTCGGCACCGGGTGTCTCTTCCGCCGCATCACCGTCTACGGCTTCGACCCGCCCAGGATCAACGTCGGCGGGCCCTGCTTCCCCAGGCTCGCCGGGCTCTTCGCCAAGACCAAGTACGAGAAGCCGAGCCTCGAGATGACAATGGCCAAGGCCAAGGCCGCGCCGGTGCCCGCCAAGGGGAAGCACGGCTTCCTGCCTCTGCCCAAGAAGACGTACGGCAAGTCGGACGCCTTCGTGGACAGCATCCCGCGCGCGTCGCACCCGTCGCCTTACGCGGCGGCGGCTGAGGGCATCGTGGCCGACGAGGCGACCATCGTGGAGGCGGTGAACGTGACGGCGGCGGCGTTCGAGAAGAAGACCGGGTGGGGCAAAGAGATCGGGTGGGTGTACGACACGGTGACGGAGGACGTGGTCACCGGGTACCGGATGCATATCAAGGGGTGGCGGTCACGCTACTGCTCCATCTACCCACACGCCTTCATCGGCACCGCACCCATCAACCTCACGGAGAGGCTCTTCCAGGTGCTCCGCTGGTCCACGGGCTCCCTCGAGATCTTCTTCTCCAAGAACAACCCGCTCTTCGGCAGCACCTACCTCCACCCGCTGCAGCGCGTCGCCTACATCAACATCACCACCTACCCCTTCACCGCCATCTTCCTCATCTTCTACACCACCGTGCCGGCGCTCTCCTTCGTCACCGGCCACTTCATCGTGCAGCGCCCCACCACCATGTTCTACGTCTACCTGGGCATCGTGCTCTCCACGCTGCTCGTCATCGCCGTGCTGGAGGTCAAGTGGGCCGGGGTCACCGTCTTCGAGTGGTTCAGGAACGGCCAGTTCTGGATGACGGCAAGTTGCTCCGCCTACCTCGCCGCCGTGTGCCAGGTGCTTACCAAGGTGATATTCCGGCGTGACATCTCCTTCAAGCTCACATCCAAGCTACCGTCGGGAGACGAGAAGAAGGACCCCTACGCCGACCTCTACGTGGTCCGCTGGACGCCGCTGATGATCACACCCATCATCATCATTTTCGTCAACATCATCGGATCCGCGGTGGCCTTCGCCAAGGTGCTGGACGGCGAGTGGACGCACTGGCTCAAGGTCGCCGGCGGGGTCTTCTTCAACTTCTGGGTGCTGTTCCACCTCTACCCGTTCGCCAAGGGCATCCTGGGGAAGCACGGCAAGACGCCAGTCGTGGTGCTCGTCTGGTGGGCATTCACCTTCGTCATCACCGCCGTGCTCTACATCAACATCCCCCACATGCATAGCTCGGGAGGCAAGCACACAACGGTGCATGGTCACCATGGCAAGAAGTTCGTCGACGCAGGGTACTACAACTGGCCGTGA

**>CslH_Svevo_2A genomic DNA**

ATGGCGGGCGGCAAGAAGCTGCACGAGAGGGTCGCCCTGGGGAGAACTGCGTGGATGCTGGCCGACTTCGTGATCCTCCTCCTCCTCCTCGCCCTCGTGGCCCGCCGCGCCGCGTCGCTCGGGGAGCGCGGCGGGACGTGGCTGGCGGCGCTCGTCTGCGAGGCGTGGTTCGCCTTCGTCTGGATCCTCAACATGAACGGCAAGTGGAGCCCCGTCCGGTTCGACACCTACCCCGAGAACCTCTCCCACAGGTACGTACGTTCTTGTGCACACTAACTGCAAAATAATGTTGACCTACAGCTTCGTGCAGCTTCTTCCTTAAACTGTGTCGTGTCTGTGATGATTTTGCTAGGCTGGAGGAGCTCCCGGCGGTGGACATGTTCGTCACGACGGCGGACCCGGCGCTGGAGCCGCCGTTGATCACGGTGAACACGGTGCTCTCGCTGCTCGCCCTGGACTACCCGGACGTCGGCAAGCTGGCGTGCTACGTCTCCGACGACGGCTGCTCCCCGGTGACGTGCTACGCGCTGCGCGAGGCCGCCAAGTTCGCCAGCCTCTGGATTCCCTTCTGCAAGAGGTATGACGTTGGTGTGAGGGCCCCTTTCATGTACTTCTCTTCCGCGCCGGAGGTTGGCACCGGTACAGCCGACCACGAGTTCCTGGAAAGCTGGGCACTCATGAAGGTTAGGCGCCATGGTGACCATTTCAGTTTCCATAATGTTTGGTCGTCCATCGTCGCCATGACCATGCATCTTCCTCGTGTACGTGTGACTTTCAGACCGAATATGAGAAGCTGGCCAGCCGGATCGAGAACGCCGACGAGGTCTCCATTCTGCGTGACGGCGGCGAAGAGTTCGCCGAGTTCATCGACGCCGAGCGCGGGAACCATCCTACCATCGTTAAGGTCGCCGCACTGACCATGTCCATGTACATCGTGTCATGCCAAACGCGTAGCAAATCCGTCTCGTGCTAATATCGTCACGGTTAACCTGTGTGAGTTCAGGTTCTCTGGGATAACAGCAAGAGCAAAGCAGGGGAAGGATTCCCACATCTGGTGTACCTCTCTCGAGAGAAAAGCCCCAGACATCGCCACAACTTCAAGGCCGGTGCCATGAATGTTCTGGTGAGCACTCTCTTGTACACAACAGTGTTTCACTGGTAATCAGTGTGTCACACAAACAGCACAATAAGTGGCAGTTGAAAGTTCAGACATGTGTACAATGCGCTTGATAATTTGCAAGCAAATAATTAAGCTGAGCGTTTCGTGGTGCAGACAAGGGTGTCGGCCGTGATGACCAACGCTCCAATCATGCTGAATGTGGACTGCGACATGTTCGCCAACAACCCGCAGGTCGCCCTGCACGCGATGTGCCTCCTGTTGGGGTTCGACGACGAGATCCACAGCGGGTTCGTCCAGGCGCCACAGAAGTTCTACGGTGGCCTCAAGGATGACCCCTTTGGCAACCAGATGCAGGTTATAACCAAGGTACTACATATGCATGTGCACAAGTGCTGTTGTGGTAGTGCACCACTAGGGTAGTGTTACAGTTGCACTGGTTTTTCTGGCATGTTCAGAAAATTGGAGGTGGGCTCGCCGGGATCCAAGGCACCTTCTACGGCGGCACGGGCTGTTTTCACCGCAGGAAGGTCATCTACGGCATGCCGCCTCCGGACACCGTCAAGCACGAGACAAGAGGTAATAAAACTGGGCACGCACAAGATGAGATCATCCGACGTAAATTGAAGTATTTGGTCAGTGCATTTCAGTTCGACTAGGGCATATCAAATGGCTGTTCTGAATTTGCCAGGTTCACCATCTTACAAGGAGCTGCAAGCCAAGTTTGGGAGCTCAAAGGAGTTGATCGAATCATCTAGGAACATCATCTCAGGGGACCTGCTCGCTAGACCAACCGTAGATATATCGAGTCGTGTCGAAATGGCAAAACAAGTAGGCGACTGCAACTATGAGGCTGGCACATGTTGGGGCCAAGAGGTGTGCTTAGCTTCGTTGCCGTATTTTTGCAGGTTTTGCTACAGTACGGCCACATCTACACACCTTCTGCAGTTTCTCTCTATTACAGTTTCTTCCATGTATTTTTGCAGATTGGGTGGGTCTATGGATCAATGACAGAGGACATTTTGACCGGTCAACGGATCCAGGCGGCGGGTTGGGAATCGGCCTTGTTGGACACCGACCCACCGGCATTCCTGGGATGTGCTCCGACCGGTGGACCAGCCAGCTTGACCCAGTTCAAGAGATGGGCAACAGGGCTTCTGGAGATACTCATCAGCCGGAACAGCCCCATCCTCGGCACCATCTTCAAGGGCCTCCAACTCCGGCAATGCCTTGGCTATCTCATCGTAGACGCGTGGCCCGTGAGGGCGCCTTTCGAGCTGTGCTATGCGCTCTTGGGACCTTTCTGCCTTCTCACAAACCAATCCTTCTTACCAACGGTACACACATTTTTGCCATGACCCATTACTACATTGCTCATAGCTGAAATTTTAGTGCATTTGCCGTTTTGCAGGCATCAGATGAAGGTTTTCACATCCCAGCGGCTCTATTTTTGACTTACAACATATACCACCTGATGGAGTACAAGGAGTGCGGGCTCTCGGTCCGCGCCTGGTGGAACAACCATAGGATGCAACGCATCACCTCGGCCTCCGCCTGGCTCCTCGCCTTCCTCACCGTCATCCTCAAGACGCTAGGGCTCTCCGAGACCGTGTTCGAGGTCACCCGCAAGGAGAGCAGCACGTCATCCGATGGCGGCGCGGGCACCGACGATGCCGATCCTGGGTTGTTCACCTTTGACTCGGCGCCCGTTTTCATCCCAGTGACGGCGCTCTCAGTGTTGAACATTGTCGCCCTCACCGTCGCGGCATGGCGCGCCGTCGTCGGGACGGTGGCGGGCGTTCATGGTGGCCCGGGCGTCGGAGAGTTCGTGTGCTGTGGCTGGATGGTGTTGTGCTTCTGGCCATTCGTGAGAGGGCTTGTCAGTAGTGGAAAGTATGGGATCCCGTGGAGTGTCAGGGTGAAGGCTGGGTTGATTGTGGCTGCGTTCGTGCACCTCTGCACAAGGAACTAA

**>CslH_Svevo_2A CDS**

ATGGCGGGCGGCAAGAAGCTGCACGAGAGGGTCGCCCTGGGGAGAACTGCGTGGATGCTGGCCGACTTCGTGATCCTCCTCCTCCTCCTCGCCCTCGTGGCCCGCCGCGCCGCGTCGCTCGGGGAGCGCGGCGGGACGTGGCTGGCGGCGCTCGTCTGCGAGGCGTGGTTCGCCTTCGTCTGGATCCTCAACATGAACGGCAAGTGGAGCCCCGTCCGGTTCGACACCTACCCCGAGAACCTCTCCCACAGGCTGGAGGAGCTCCCGGCGGTGGACATGTTCGTCACGACGGCGGACCCGGCGCTGGAGCCGCCGTTGATCACGGTGAACACGGTGCTCTCGCTGCTCGCCCTGGACTACCCGGACGTCGGCAAGCTGGCGTGCTACGTCTCCGACGACGGCTGCTCCCCGGTGACGTGCTACGCGCTGCGCGAGGCCGCCAAGTTCGCCAGCCTCTGGATTCCCTTCTGCAAGAGGTATGACGTTGGTGTGAGGGCCCCTTTCATGTACTTCTCTTCCGCGCCGGAGGTTGGCACCGGTACAGCCGACCACGAGTTCCTGGAAAGCTGGGCACTCATGAAGACCGAATATGAGAAGCTGGCCAGCCGGATCGAGAACGCCGACGAGGTCTCCATTCTGCGTGACGGCGGCGAAGAGTTCGCCGAGTTCATCGACGCCGAGCGCGGGAACCATCCTACCATCGTTAAGGTTCTCTGGGATAACAGCAAGAGCAAAGCAGGGGAAGGATTCCCACATCTGGTGTACCTCTCTCGAGAGAAAAGCCCCAGACATCGCCACAACTTCAAGGCCGGTGCCATGAATGTTCTGACAAGGGTGTCGGCCGTGATGACCAACGCTCCAATCATGCTGAATGTGGACTGCGACATGTTCGCCAACAACCCGCAGGTCGCCCTGCACGCGATGTGCCTCCTGTTGGGGTTCGACGACGAGATCCACAGCGGGTTCGTCCAGGCGCCACAGAAGTTCTACGGTGGCCTCAAGGATGACCCCTTTGGCAACCAGATGCAGGTTATAACCAAGAAAATTGGAGGTGGGCTCGCCGGGATCCAAGGCACCTTCTACGGCGGCACGGGCTGTTTTCACCGCAGGAAGGTCATCTACGGCATGCCGCCTCCGGACACCGTCAAGCACGAGACAAGAGGTTCACCATCTTACAAGGAGCTGCAAGCCAAGTTTGGGAGCTCAAAGGAGTTGATCGAATCATCTAGGAACATCATCTCAGGGGACCTGCTCGCTAGACCAACCGTAGATATATCGAGTCGTGTCGAAATGGCAAAACAAGTTTTGCTACAGTACGGCCACATCTACACACCTTCTGCAGTTTCTCTCTATTACAGTTTCTTCCATGTATTTTTGCAGATTGGGTGGGTCTATGGATCAATGACAGAGGACATTTTGACCGGTCAACGGATCCAGGCGGCGGGTTGGGAATCGGCCTTGTTGGACACCGACCCACCGGCATTCCTGGGATGTGCTCCGACCGGTGGACCAGCCAGCTTGACCCAGTTCAAGAGATGGGCAACAGGGCTTCTGGAGATACTCATCAGCCGGAACAGCCCCATCCTCGGCACCATCTTCAAGGGCCTCCAACTCCGGCAATGCCTTGGCTATCTCATCGTAGACGCGTGGCCCGTGAGGGCGCCTTTCGAGCTGTGCTATGCGCTCTTGGGACCTTTCTGCCTTCTCACAAACCAATCCTTCTTACCAACGGCATCAGATGAAGGTTTTCACATCCCAGCGGCTCTATTTTTGACTTACAACATATACCACCTGATGGAGTACAAGGAGTGCGGGCTCTCGGTCCGCGCCTGGTGGAACAACCATAGGATGCAACGCATCACCTCGGCCTCCGCCTGGCTCCTCGCCTTCCTCACCGTCATCCTCAAGACGCTAGGGCTCTCCGAGACCGTGTTCGAGGTCACCCGCAAGGAGAGCAGCACGTCATCCGATGGCGGCGCGGGCACCGACGATGCCGATCCTGGGTTGTTCACCTTTGACTCGGCGCCCGTTTTCATCCCAGTGACGGCGCTCTCAGTGTTGAACATTGTCGCCCTCACCGTCGCGGCATGGCGCGCCGTCGTCGGGACGGTGGCGGGCGTTCATGGTGGCCCGGGCGTCGGAGAGTTCGTGTGCTGTGGCTGGATGGTGTTGTGCTTCTGGCCATTCGTGAGAGGGCTTGTCAGTAGTGGAAAGTATGGGATCCCGTGGAGTGTCAGGGTGAAGGCTGGGTTGATTGTGGCTGCGTTCGTGCACCTCTGCACAAGGAACTAA

**>CslH_Svevo_2B genomic DNA** ATGGCGGGCGGCAAGAAGCTGCAGGAGAGGGTCGCCCTGGGCAGGAGTGCGTGGATGCTGGCCGACTTCGTGATCCTCTTCCTCCTCCTCGCCCTCGTGGCCCGCCGCGCCGCGTCGCTCGGGGAGCGCGGCGGGACGTGGCTGGCAGCGCTCGTCTGCGAGGCGTGGTTCGCCTTCGTGTGGATCCTCAACATGAACGGCAAGTGGAGCCCCGTCCGGTTCGACACCTACCCCGACAACCTCTCCCACAGGTACGTACGTTCTTGTGCACACTAACTGCAAAATAATGTTGACCTACAACTTCGTGCAACTTCTTCCTTAAACTGTGTCGTGTCTGTGATGATTTTGCTAGGATGGAGGAGCTCCCAGCGGTGGACATGTTCGTCACGACGGCGGACCCGGCGCTGGAGCCGCCGTTGATCACGGTGAACACGGTGCTCTCGCTGCTCGCCCTGGACTACCCGCACGTCGGCAAGCTGGCGTGCTACGTCTCCGACGACGGCTGCTCCCCCTTGACGTGCTACGGTCTGCACGAGGCCGCCAAGTTCGCCAGCCTCTGGGTTCCCTTCTGCAAGAGGCACGACGTTGGTGTGAGGGCCCCTTTCATGTACTTCTCTTCCGCGCCGGAGGTTGACACCGGTACAGTCGACCACGAGTTCCTGGAAAGCTGGGCACTCATGAAGGTCAGCCGATGATGATGATGTCAGTTTCCATAATGTTTGGTCGTCCATCATCGCCATGACCATGCATCTTCCTTGTGTACGTGTGACTTTCAGAGCGAATATGAGAAGCTGGCCAGCCGGATCGAGAACGCCGACGAGGTCTCCATTCTGCGTGACGGCGGCGACGATTTCGCCGAGTTCATTGACGCCGAGCGCGGGAACCATCCTACCATCGTTAAGGTCGCTGAACTGACCATATCCACGTGTCCATGTACATCGTGTCGTGCCAAACGCATAGCGAATCCGTCTCGTGCTAATATCGTCACGGTTAACCTGTCTGAGTTCAGGTTCTCTGGGATAACAGCAAGAACAAAACAGGTGAAGGATTCCCACATCTGGTGTACCTCTCGAGAGAGAAAAGCCCCAGACATCGTCACAACTTTAAGGCCGGTGCCATGAATGTTCTGGTGAGCACTCTCTTCTACTCAATACAGTGTTGCACTACTAATCAGTGTGTCACACAAACAGCAAAAAGTGGCAGATAAAAGCTCAGACGGTTGCACGACACATTTGATACTAATTAAGCTGAGCATTTCGTGGTGCAGACAAGGGTGTCGGCCGTGATGACCAACGCTCCGATCATGCTGAATGTGGACTGCGACATGTTTGCCAACAACCCGCAGGTCGCCCTACACGCGATGTGCCTCCTGTTGGGGTTCGACGACGAGATCCACAGCGGGTTCGTCCAGGCGCCACAGAAGTTCTACGGTGGCCTCAAGGATGACCCCTTTGGCAACCAGATGCAGGTTATAACCAAGGTACTACATATGCATGTGCACAAGTGCTGTTGTGGTAGTGCACCACTAGGGTAGTGTTACAGTTGCACTGGTTTTTCTGGCATGTTCAGAAAATTGGAGGTGGGCTCGCCGGGATCCAAGGCACGTTCTACGGCGGCACGGGCTGTTTTCACCGCAGGAAGGTCATTTACGGCATGCCGCCTCCGGACACCGTCAAGCACGAGACAAGAGGTAATAAAACTGGGCACACAAAAGATGAGGCATCCGGCGTAAATTGGAGTATTTGGCCAGTGCATTTCAGTTCGACTAGGGCATATCAAATGGCTTTCTGAATTTGCCAGGTTCACCATCTTACAAGGAGCTGCAAGCCAAGTTTGGGAGCTCAAAGGAGTTGATCGAATCATCTAGGAACATCATCTCAGGAGACCTGCTCGCTAGACCAACCGTAGATATATCAAGTCGGGTCGAAATGGCAAAACAAGTAGGCGACTGCAACTATGAGGCTGGCACATATTGGGGCCAAGAGGTGTGCTTAGCTTCGTTGCCGTATTTTTGCAGGTTTTGCTACAGTACGGCCACATCTACAAACCTTCTGCAGTTTCTCTCTATTACAGTTTCTTCCATCTATTTTTGCAGATTGGGTGGGTCTATGGATCAATGACAGAGGACATTTTGACCGGGCAACGGATCCAAGCGGCGGGTTGGAAATCGGCCTTGTTGGACACCGACCCACCGGCATTCTTGGGATGTGCTCCGACAGGGGGGCCGGCTAGCTTGACCCAGTTCAAGAGATGGGCAACAGGGCTTCTGGAGATACTCATCAGCCGGAACAGCCCCATCCTCGGCACCATCTTCAGGCGCCTCCAACTCCGGCAATGCCTTGCCTATCTCATCGTCAACGCGTGGCCCATGAGGGCACCTTTCGAGATGTGTTACGCGCTATTGGGACCTTTCTGCCTTCTCACAAACCAGTCCTTCTTGCCAACGGTACACACATTTTTGCCATGACCCTTTACTACATTGCTCATAGCTGAAATTTCAGTACACGTGGTGATGTGGAAACACAAGTCTATGCAACTAAACAAAAATGTTTGTGTAATTTGTTTGATAAGATTTGTGCATTTGCTGTTTTGCAGACATCTAATGAAGGTTTTCGCATCCCAGCGGCTCTATTCTTGAGTTACCACGTATACCACCTGATGGAGTACAAGGAGTGCGGGCTCTCGGTCCGCGCCTGGTGGAACAACCACAGGATGCAACGCATCACCTCGGCCTCCGCCTGGCTCCTCGCCTTCCTCACCGTCATCCTCAAGACGCTAGGGCTCTCCGAGACCGTGTTCGAGGTCACCCGCAAGGAGAGCAGCACGTCCTCCGATGGTGGCGCGGGCACCGACGAGGCCGATACTGGGCTGTTCACCTTCGACTCGGCGCCCGTTTTCATCCCGGTGACGGCGCTCTCAATGCTGAACATTGTCGCCCTCGCCGTCGCGGCATGGCGCGCCGTTGTCGGGACGGCGGCGGGCGTTCATGGTGGCCCGGGAGTCGGAGAGTTCGTGTGCTGTGGCTGGATGGTGCTGTGCTTCTGGCCGTTCATGAGAGGGCTTGTCAGCAGTGGAAAGTATGGGATCCCGTGGAGTGTCAGGGTGAAGGCTGGGTTGATTGTGGCTGCGTTCGTGCACCTCTGCACAAGGAACTAA

**> CslH_Svevo_2B CDS**

ATGGCGGGCGGCAAGAAGCTGCAGGAGAGGGTCGCCCTGGGCAGGAGTGCGTGGATGCTGGCCGACTTCGTGATCCTCTTCCTCCTCCTCGCCCTCGTGGCCCGCCGCGCCGCGTCGCTCGGGGAGCGCGGCGGGACGTGGCTGGCAGCGCTCGTCTGCGAGGCGTGGTTCGCCTTCGTGTGGATCCTCAACATGAACGGCAAGTGGAGCCCCGTCCGGTTCGACACCTACCCCGACAACCTCTCCCACAGGATGGAGGAGCTCCCAGCGGTGGACATGTTCGTCACGACGGCGGACCCGGCGCTGGAGCCGCCGTTGATCACGGTGAACACGGTGCTCTCGCTGCTCGCCCTGGACTACCCGCACGTCGGCAAGCTGGCGTGCTACGTCTCCGACGACGGCTGCTCCCCCTTGACGTGCTACGGTCTGCACGAGGCCGCCAAGTTCGCCAGCCTCTGGGTTCCCTTCTGCAAGAGGCACGACGTTGGTGTGAGGGCCCCTTTCATGTACTTCTCTTCCGCGCCGGAGGTTGACACCGGTACAGTCGACCACGAGTTCCTGGAAAGCTGGGCACTCATGAAGAGCGAATATGAGAAGCTGGCCAGCCGGATCGAGAACGCCGACGAGGTCTCCATTCTGCGTGACGGCGGCGACGATTTCGCCGAGTTCATTGACGCCGAGCGCGGGAACCATCCTACCATCGTTAAGACAAGGGTGTCGGCCGTGATGACCAACGCTCCGATCATGCTGAATGTGGACTGCGACATGTTTGCCAACAACCCGCAGGTCGCCCTACACGCGATGTGCCTCCTGTTGGGGTTCGACGACGAGATCCACAGCGGGTTCGTCCAGGCGCCACAGAAGTTCTACGGTGGCCTCAAGGATGACCCCTTTGGCAACCAGATGCAGGTTATAACCAAGAAAATTGGAGGTGGGCTCGCCGGGATCCAAGGCACGTTCTACGGCGGCACGGGCTGTTTTCACCGCAGGAAGGTCATTTACGGCATGCCGCCTCCGGACACCGTCAAGCACGAGACAAGAGGTTCACCATCTTACAAGGAGCTGCAAGCCAAGTTTGGGAGCTCAAAGGAGTTGATCGAATCATCTAGGAACATCATCTCAGGAGACCTGCTCGCTAGACCAACCGTAGATATATCAAGTCGGGTCGAAATGGCAAAACAAGTAGGCGACTGCAACTATGAGGCTGGCACATATTGGGGCCAAGAGACATCTAATGAAGGTTTTCGCATCCCAGCGGCTCTATTCTTGAGTTACCACGTATACCACCTGATGGAGTACAAGGAGTGCGGGCTCTCGGTCCGCGCCTGGTGGAACAACCACAGGATGCAACGCATCACCTCGGCCTCCGCCTGGCTCCTCGCCTTCCTCACCGTCATCCTCAAGACGCTAGGGCTCTCCGAGACCGTGTTCGAGGTCACCCGCAAGGAGAGCAGCACGTCCTCCGATGGTGGCGCGGGCACCGACGAGGCCGATACTGGGCTGTTCACCTTCGACTCGGCGCCCGTTTTCATCCCGGTGACGGCGCTCTCAATGCTGAACATTGTCGCCCTCGCCGTCGCGGCATGGCGCGCCGTTGTCGGGACGGCGGCGGGCGTTCATGGTGGCCCGGGAGTCGGAGAGTTCGTGTGCTGTGGCTGGATGGTGCTGTGCTTCTGGCCGTTCATGAGAGGGCTTGTCAGCAGTGGAAAGTATGGGATCCCGTGGAGTGTCAGGGTGAAGGCTGGGTTGATTGTGGCTGCGTTCGTGCACCTCTGCACAAGGAACTAA
